# Modelling heme-mediated brain injury associated with cerebral malaria in human brain cortical organoids

**DOI:** 10.1038/s41598-019-55631-8

**Published:** 2019-12-16

**Authors:** Adriana Harbuzariu, Sidney Pitts, Juan Carlos Cespedes, Keri Oxendine Harp, Annette Nti, Andrew P. Shaw, Mingli Liu, Jonathan K. Stiles

**Affiliations:** 1 Department of Microbiology, Biochemistry and Immunology, Morehouse School of Medicine, 720 Westview Dr, Atlanta, GA 30310 USA; 2 Parker H. Petit Institute for Bioengineering and Bioscience, Georgia Institute of Technology, 315 Ferst Drive, Atlanta, GA 30332 USA

**Keywords:** Induced pluripotent stem cells, Malaria

## Abstract

Human cerebral malaria (HCM), a severe encephalopathy associated with *Plasmodium falciparum* infection, has a 20–30% mortality rate and predominantly affects African children. The mechanisms mediating HCM-associated brain injury are difficult to study in human subjects, highlighting the urgent need for non-invasive *ex vivo* human models. HCM elevates the systemic levels of free heme, which damages the blood-brain barrier and neurons in distinct regions of the brain. We determined the effects of heme on induced pluripotent stem cells (iPSCs) and a three-dimensional cortical organoid system and assessed apoptosis and differentiation. We evaluated biomarkers associated with heme-induced brain injury, including a pro-inflammatory chemokine, CXCL-10, and its receptor, CXCR3, brain-derived neurotrophic factor (BDNF) and a receptor tyrosine-protein kinase, ERBB4, in the organoids. We then tested the neuroprotective effect of neuregulin-1 (NRG-1) against heme treatment in organoids. Neural stem and mature cells differentially expressed CXCL-10, CXCR3, BDNF and ERBB4 in the developing organoids and in response to heme-induced neuronal injury. The organoids underwent apoptosis and structural changes that were attenuated by NRG-1. Thus, cortical organoids can be used to model heme-induced cortical brain injury associated with HCM pathogenesis as well as for testing agents that reduce brain injury and neurological sequelae.

## Introduction

Brain organoids are self-assembled three-dimensional (3D) aggregates derived from pluripotent stem cells (iPSCs) with cell types and formations that mimic the embryonic human brain^1–7^. They have emerged as innovative model systems that can be used to investigate human brain development, neurologic disease pathogenesis and drug development^8–10^. Human neurodevelopmental disorders, disease-induced neuronal injury, cognitive functions and new interventions can now be investigated using iPSCs obtained by reprogramming somatic cells that maintain the genetic background of their respective hosts. iPSCs pluripotent capacity has been used to model early fetal brain development, eliminating ethical concerns associated with the use of embryonic stem cells. These unique features have enabled the exploration of complex diseases, including schizophrenia^11^ and Timothy syndrome^12^. Whole brain organoids have been developed to study Zika-associated microcephaly^3^, while cortical organoids have been used to model lissencephaly, Zika infection, Autism Spectrum Disorder (ASD) and brain evolution^13,14^. While significant progress has been made in utilizing organoids for modelling organogenesis and developmental disorders, the neuropathogenesis of infectious parasitic diseases, including cerebral malaria^15^, trypanosomiasis^16^, cysticercosis^17^ and toxoplasmosis^18^, which are responsible for significant neurological disorders and cognitive dysfunctions throughout the world, has not been modelled^19^.

Malaria causes more than 0.5 million deaths annually, with HCM and severe malaria anemia being major causes of death in sub-Saharan African children. HCM pathogenesis is complex and multi-factorial and involves the sequestration of infected red blood cells in brain microvessels, systemic host inflammatory response, and hemostasis dysfunction. The role of HCM-induced hemolysis, methemoglobin and free heme in HCM pathogenesis was recently highlighted in mice and in cross-sectional studies in humans^20^. HCM increased hemolysis due to intra-erythrocytic (iRBC) parasite proliferation results in blood-brain barrier (BBB) dysfunction, release of free heme into the brain parenchyma, widespread edema, petechial hemorrhages, neuronal injury, hemiparesis in young adults^21^ and death^22^.

We and others identified, through the analysis of cerebrospinal fluid (CSF) of Ghanaian children dying of HCM, that CXCL-10 has pro-apoptotic effects on brain neurons and glia. Comparative studies in India confirmed that CXCL-10 is elevated in HCM non-survivors compared to healthy controls, patients with mild malaria and HCM survivors^23^. Further analysis of specificity and sensitivity by receiver operating characteristic (ROC)^24^ curves revealed that CXCL-10 can be used as a functional biomarker of HCM mortality. The genetic deletion of *CXCL-10* (*CXCL-10-/-*) or of its receptor *CXCR3* (*CXCR3-/-*) in experimental cerebral malaria (ECM) studies involving *Plasmodium berghei* ANKA infections attenuates ECM pathogenesis and mortality. Others have reported heme-induced morphological and functional changes in astrocytes in ECM pathogenesis^25^. Furthermore, in human HCM^26,27^, murine ECM^28^ and *in vitro* models^29^, increased malaria-induced free heme has been shown to elevate the levels of CXCL-10, brain-derived neurotrophic factor (BDNF) and other factors that are tightly correlated with brain injury.

To mitigate the deleterious effects of HCM, various clinical trials involving erythropoietin (EPO)^30^, amodiaquine-artesunate^31^, dihydroartemisinin (DHA)-piperaquine^32^, curdlan sulfate^33^, pentoxifylline^34^ and intravenous immunoglobulin^35^ have been conducted with mixed results and various side effects. These failures may have been due to the lack of a suitable *ex vivo* model for pre-clinical screening of these drugs. Recently, NRG-1, a member of the *EGF* family of encoded genes located on chromosome 8, has been used to attenuate ECM-induced brain injury and mortality in mice^36^. The infusion of recombinant NRG-1 activates the NRG-1/ERBB4 signaling pathway and attenuates ischemia/reperfusion-induced brain injury^29^. NRG-1 has also been used to attenuate acute ischemic stroke, traumatic brain injury and nerve agent poisoning^37–39^. However, the cytoprotective and neuroprotective properties of NRG-1 have not been evaluated in brain organoid models. Until now, *in vitro* 2D cultures, animal models and post-mortem human subjects with various limitations have been employed to investigate HCM pathogenesis, brain injury and interventions^20,40–43^. The assessment of HCM-associated brain injury in human post-mortem tissues has provided limited cross-sectional data with limited insight into HCM pathogenesis.

To overcome limitations in our understanding of cerebral malaria-associated brain injury mechanisms, there is an urgent need for experimental models that recapitulate the complexity and organization of the human brain and are amenable to manipulation by current non-invasive molecular technologies. Such a model can be used to investigate human brain development, neurologic disease pathogenesis and drug development associated with HCM in a non-invasive way.

In this study, an induced pluripotent cell line obtained by reprogramming CD34^+^ human umbilical cord blood cells was used to develop brain cortical organoids. Next, we investigated the feasibility of using 3D iPSC-derived forebrain structures as a model to assess the direct effects of heme, a by-product of malaria-induced hemolysis, on human brain development, structure and key functional biomarkers. We also tested the hypothesis that NRG-1 attenuates heme-induced human brain cortical organoid injury, as observed in the ECM model. We propose this *ex vivo* human brain model as a viable alternative for studies related to heme-induced brain injury associated with HCM, traumatic brain injury, stroke, and sickle cell disease.

## Results

### iPSCs characterization

**Figure 1:**
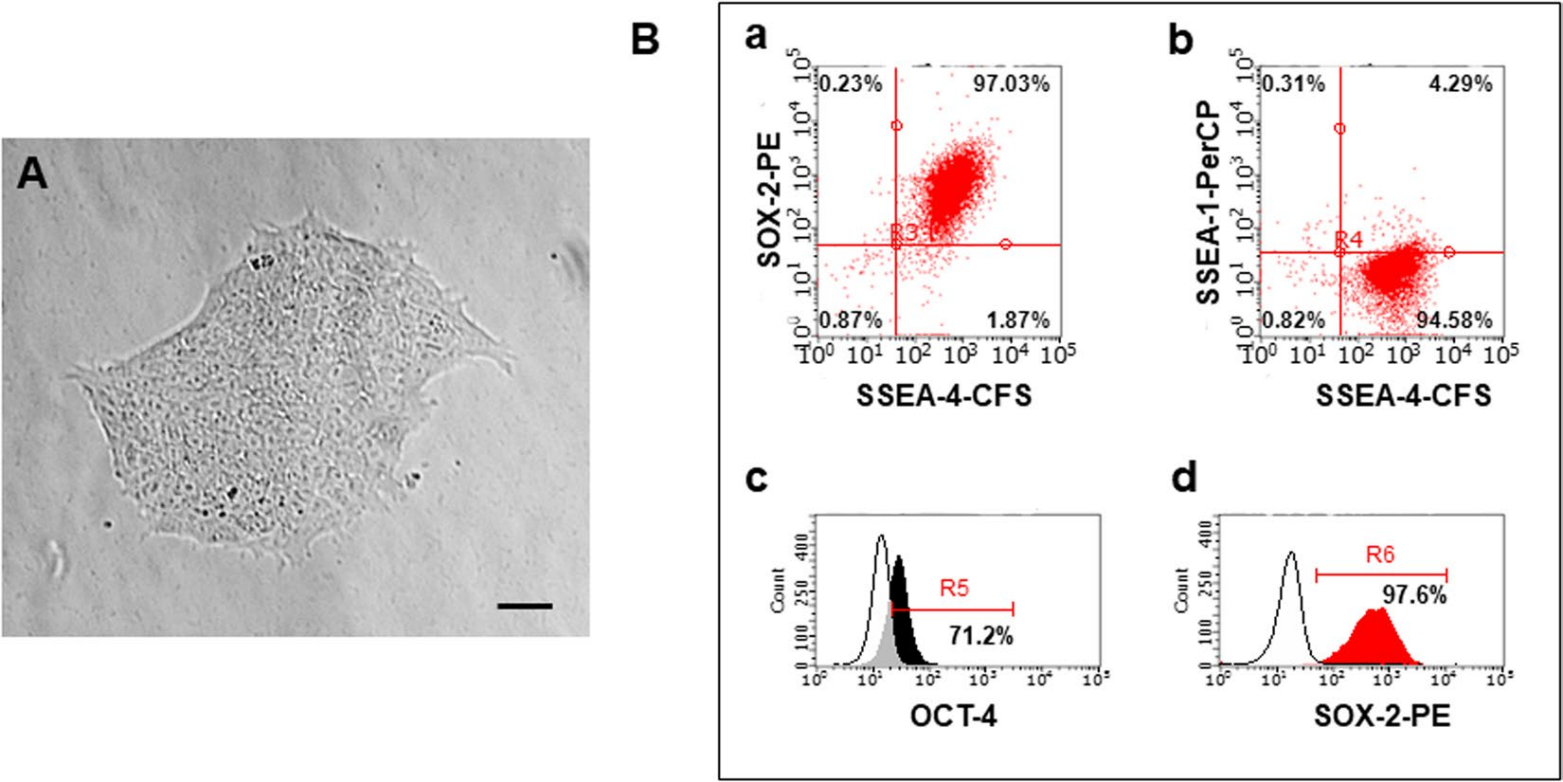
Cultivation and authentication of human umbilical cord-derived iPSCs. (**A**) Human iPSC colonies (passage 4) with smooth edges and a non-differentiated phenotype. Magnification: 10x. Scale bar: 80 µm. (**B**) Confirmation of human iPSC pluripotency (passage 3) by multi-colour flow cytometry. The majority of cells expressed the pluripotent markers (a,d) SOX-2, (b) SSEA-4 and (c) OCT-3/4 and were negative for (b) SSEA-1.

Previous studies have reported that iPSCs can be obtained from various sources, namely skin fibroblasts, peripheral blood mononuclear cells (PBMCs), urine and cord blood. We characterized the morphology and phenotype of CD34^+^ umbilical cord blood-derived iPSCs to ensure consistent and reproducible results in our experiments. To confirm the undifferentiated state of iPSCs, their morphology was assessed as previously described^8^. The iPSC colonies (Fig. 1A) were compact and round with smooth edges, while the cells appeared dense, small and had a high nucleus: cytoplasm ratio. For clinical-grade iPSC quality testing, requirements include characterization of a minimum of two markers; >70% of cells should be positive^44^. Using flow cytometry (Fig. 1B), we assessed the expression of pluripotency markers {stage-specific embryonic antigen-4 (SSEA-4)^45^, sex determining region Y box-2 (SOX-2)^46^, and OCTamer-binding transcription factor 3/4 (OCT3/4)}^47^ at the single-cell level and accounted for the homogeneity of the induced pluripotent cell line^48^. Human embryonic stem cells (ESCs) are characterized by low levels of SSEA-1 (CD15) expression^49,50^. SSEA-1 increases after ESC differentiation towards neuronal stem cells (NSCs)^51–53^. Our results showed that 97.03% of cells were positive for both SOX-2 and SSEA-4 (Fig. 1Ba,d), while 71.2% were positive for OCT 3/4 (Fig. 1Bc) and only 4.6% were positive for SSEA-1 (Fig. 1Bb).

### NRG-1 attenuates heme-induced iPSCs apoptosis

**Figure 2:**
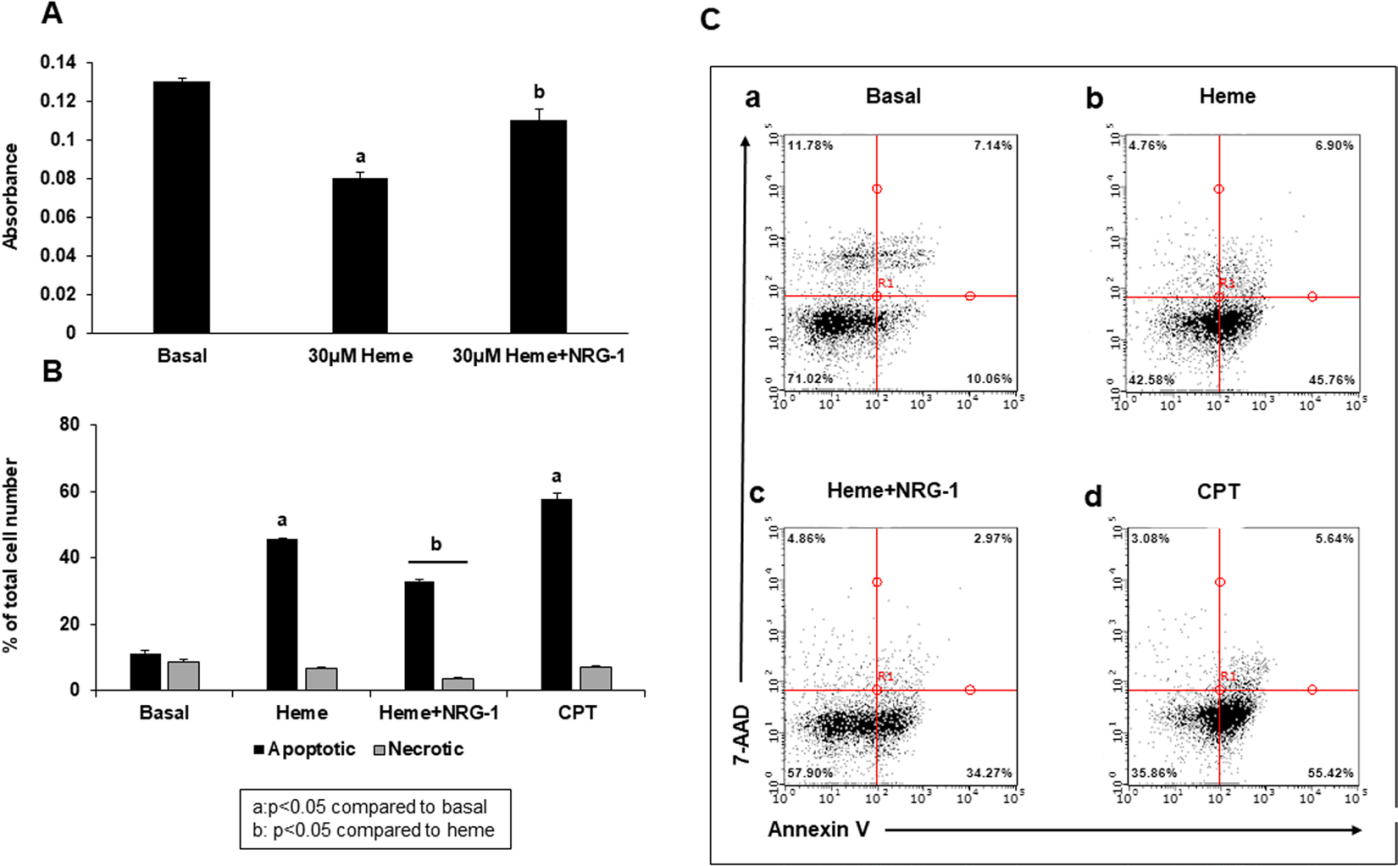
Heme-induced apoptosis of iPSCs is diminished by NRG-1. iPSCs were treated with heme for 8 hours, and NRG-1 was then added for 18 hours. Cell viability was determined using the CCK-8 assay (**A**), while the apoptotic/necrotic cell number was assessed using flow cytometry analysis (**B**,**C**). (**A**) Cell viability decreased significantly when iPSCs were treated with heme. NRG-1 reduced the effects of heme. (**B**) The number of apoptotic cells increased with heme compared to basal conditions. The addition of NRG-1 to heme treatment decreased not only the number of apoptotic but also the number of necrotic cells. CPT was used as a positive control for apoptosis. The data shown are the mean values + standard error (SE) (n = 3 experiments) (a: p < 0.05 compared to basal control; b: p < 0.05 compared to heme). (**C**) Representative Annexin V/7-AAD flow cytometry results for cells undergoing apoptosis and necrosis under heme treatment. The percentage of cells in each quadrant is shown. Taken together, these results show that heme induced iPSC injury, while the addition of NRG-1 reduced this effect by reducing the apoptotic/necrotic cell number, thus increasing the number of live cells.

We previously demonstrated^54^ that 30 μM heme (clinically relevant concentration in HCM patients) induces apoptosis in endothelial, neuronal, glial and BeWo trophoblast cells, which is attenuated by NRG-1^54^. The IC50 for heme in iPSCs was 38 µM (data not shown), consistent with previous results in other cell lines. Camptothecin (CPT) was used as a positive control for iPSCs apoptosis [IC50 = 0.41 µM (data not shown)]. Based on a previous study, in which a dose of 100 ng/ml NRG-1 rescued 50% of apoptotic cells^29^, we next used this dose in combination with heme. Based on the CCK-8 assay for toxicity, 30 μM heme reduced cell viability by approximately 40%, whereas the addition of NRG-1 significantly attenuated the effects of heme by 23% (Fig. 2A). The assessment of heme-induced iPSCs apoptosis using Annexin V assay showed that heme significantly increased the percentage of early apoptotic cells by more than 4-fold compared to basal levels, while the percentage of necrotic cells remained unchanged. Cell viability significantly improved when 100 ng/ml NRG-1 was added to heme treatment; the percent of early apoptotic cells decreased by 25% compared to that observed with heme treatment, while the percent of necrotic cells decreased from 6.62% to 3.5% (3.12% represents 47% cell survival). CPT, as expected, induced apoptosis (57.6%) and necrosis (7.1%) (Fig. 2B) of iPSCs. A representative apoptotic assay indicated decreased viability following heme treatment and subsequent attenuation of this effect following the addition of NRG-1 (Fig. 2C).

### Effects of heme on iPSCs pluripotency

**Figure 3:**
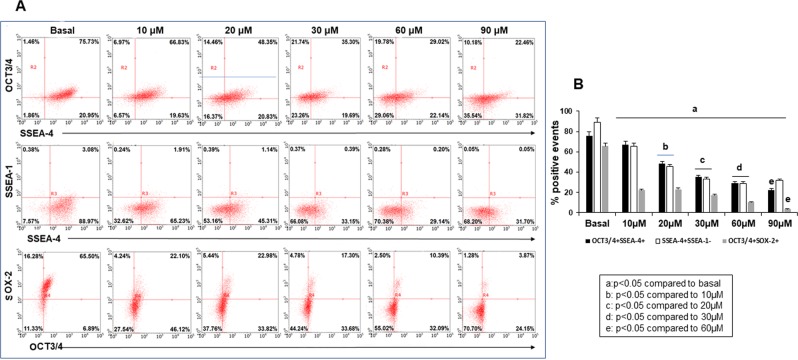
Heme induces iPSC differentiation. iPSCs were treated with heme for 18 hours. (**A**) The number of cells positive for pluripotency markers (OCT-3/4, SSEA-4, and SOX-2) was determined using flow cytometry analysis. (**B**) The number of pluripotent cells decreased with increasing heme concentrations. The lowest percent of pluripotent cells was observed when heme was used at a concentration of 90 μM.

iPSCs have the capacity to self-renew and undergo differentiation in response to specific growth factors in culture. To determine the effect of heme on the pluripotent state of iPSCs, we quantitatively assessed the expression of pluripotency markers using flow cytometry analysis (Fig. 3). Heme-induced spontaneous iPSCs differentiation was assessed by growing iPSCs to 70–80% confluence in 6-well plates coated with Geltrex and incubating them with increasing doses of heme (0. 10, 20, 30, 60 and 90 µM) for 18 hours. Analysis of the expression of pluripotency markers (OCT3/4, SOX-2 and SSEA-4) showed that heme treatment reduced the number of pluripotent cells. In addition, the percent of cells that were negative for pluripotnt stem cell markers increased (Fig. 3A). Compared to no treatment, all heme treatment doses reduced the number of OCT3/4^+^SSEA-4^+^, SSEA-4^+^SSEA-1^−^ and SOX-2^+^OCT3/4^+^ iPSCs. As the heme treatment doses increased, the percent of pluripotent cells decreased compared to basal and lower heme treatment doses (Fig. 3B). Thus, the data demonstrate that heme-induced spontaneous iPSC differentiation occurs with increasing heme concentrations.

### Heme alters the expression of ERBB4 and endogenous NRG-1 in iPSCs

**Figure 4:**
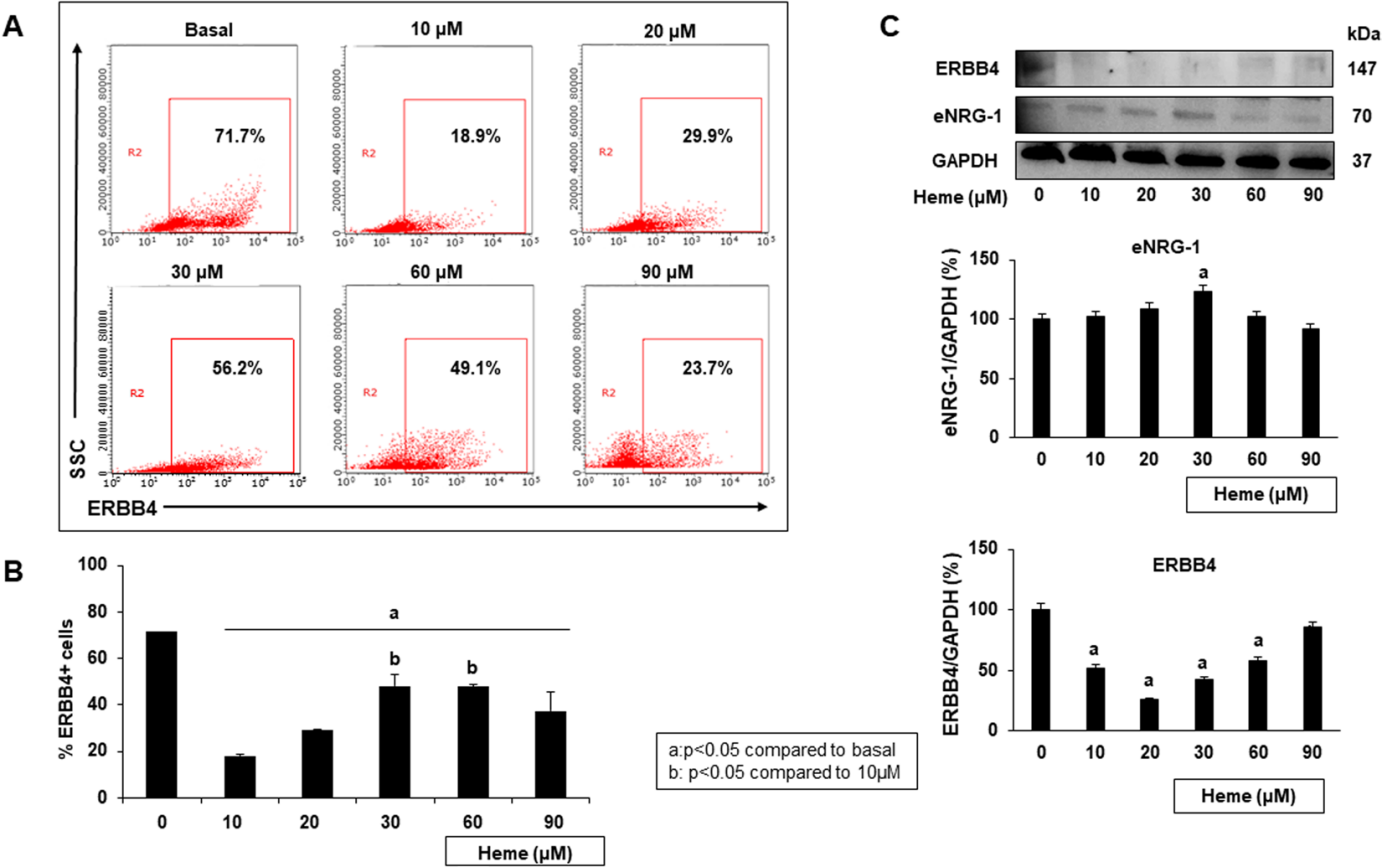
Heme treatment affects ERBB4 and NRG-1 expression in iPSCs. iPSCs were treated with heme for 18 hours. The number of cells positive for ERBB4 was assessed using flow cytometry analysis. The ERBB4 and endogenous NRG-1 (eNRG-1) protein expression levels were determined using Western blotting. (**A**) Representative flow cytometry results illustrating the percent of iPSCs that express ERBB4. (**B**) The number of cells positive for ERBB4 increased as the heme-induced injury level increased. (**C**) Cell lysates from iPSCs treated with various heme doses were immunoblotted for ERBB4 and NRG-1. Western blot densitometry analysis was performed using the ImageJ program (full blots are shown in the Supplementary Information file, Supplementary Fig. S1). The results show that NRG-1 expression levels increased as the heme concentration increased, while ERBB4 expression decreased in a similar manner (a: p < 0.05 compared to basal conditions; b: p < 0.05 compared to 10 µM treatment).

We previously reported that NRG-1 expression increases with disease severity in the cortices of ECM mice, while ERBB4 expression decreases^29^. However, the expression of NRG-1 and ERBB4 has not been previously determined in embryonic stem cells. The number of iPSCs that expressed ERBB4 under increasing heme concentrations was assessed using flow cytometry (Fig. 4A,B). Compared to basal conditions, heme induced a significant decrease in the number of ERBB4-expressing cells. However, at lower heme concentrations (10 and 20 μM), most of the cells (82.1% and 70.9%, respectively) did not express ERBB4. As heme-induced injury increased, the number of ERBB4-expressing iPSCs increased significantly compared to that under low-heme conditions. Next, we assessed whether ERBB4 expression in iPSCs correlates with the severity of heme-induced injury and observed similar trends as those described above. For example, at 30 μM heme, 52% of the iPSCs were ERBB4^+^, while the levels of ERBB4 expression were 58% less than those observed under untreated conditions. The levels of endogenous NRG-1 at 30 μM heme were significantly higher than those observed under basal conditions (Fig. 4C). Interestingly, in iPSCs, an increase in heme dose did not result in an increase in endogenous NRG-1 levels.

### Cortical organoids express ERBB4 and NRG-1

**Figure 5:**
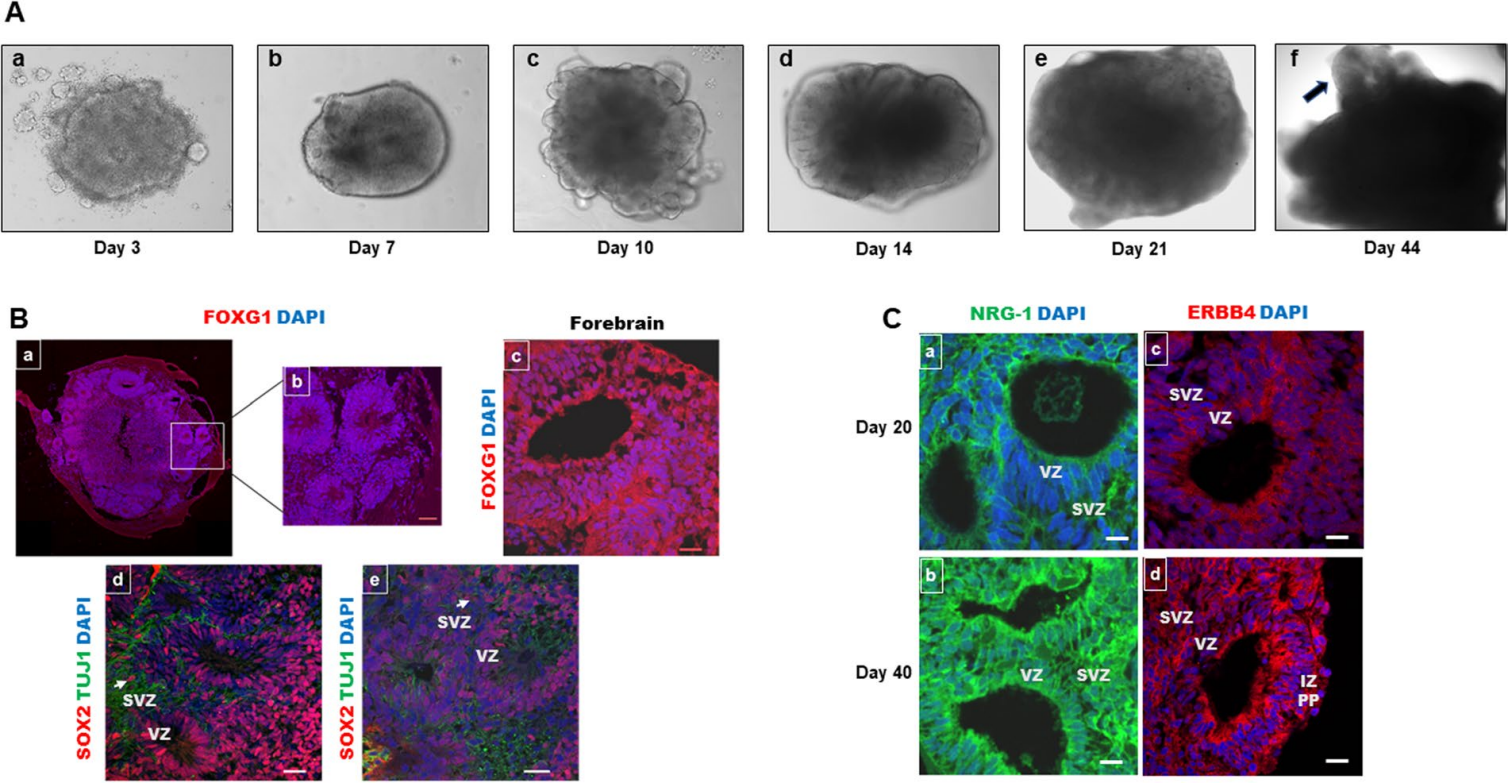
Human cortical organoids express ERBB4 and NRG-1. (**A**) The development of human cortical organoids. (a) Embryoid bodies. (b) Induction towards a neuronal fate. (c) The development of neuroepithelia. (d,e). Organoid maturation. (f) Forebrain structure within organoids (arrow) becomes evident after 40 days. (**B**) Representative sections of whole 20-day cortical organoids (a) and the inside of (b) cortical organoids stained for FOXG1 showing forebrain identity with structures displayed around the organoid core. At 40 days (c), FOXG1 staining was similar. SOX-2 (a neural stem cell marker) (red) and TUJ1 (a neuronal specific marker) (green) staining in cortical organoids at 20 (d) and 40 (e) days. (**C**) Cortical organoids expressed NRG-1 (a,b) and ERBB4 (c,d) at 20 and 40 days, respectively. Scale bar: 20 µm.

During human neocortical development, neuroepithelial cells (NECs) comprising the neural tube begin to organize in areas around a cavity-like structure called the ventricular zone (VZ). Radial glial (RG) cells make up a major population of neural stem cells that occupy the proliferative VZ^55^. The next layers that form basally from the VZ during human embryogenesis are the subventricular zone (SVZ), which has two distinct proliferative zones [the inner SVZ (ISVZ) and the outer SVZ (OSVZ)], the intermediate zone (IZ) and the cortical plate (CP). Another population of neural stem/progenitor cells, which is composed of intermediate progenitor cells (IPCs) and outer radial glial (oRG) cells, has recently been reported to occupy the SVZ^56^. As fetal development progresses, more neurons are generated in the VZ and SVZ (especially the OSVZ), migrate to the IZ and CP and eventually develop the adult cortex^48^. Studies have shown that RG cells derived from human midgestational brain tissue are direct parental cells of forebrain neurons and astrocytes^55,56^. During human fetal cortex development, at 5–6 gestation weeks (GW), young neurons form a primordial plexiform layer (PPL); after 2 weeks (7–8 GW), the CP emerges. At midgestation (20 GW), the bulk of neurogenesis in the human cerebral cortex has occurred, and RG cells begin transforming into GFAP+ astrocytes in the IZ and in the CP^57^. Recently, the generation of neural organoids from iPSCs has been an important tool for development studies^3^. Unlike 2D cell culture and mouse models, organoids dynamically resemble human brain developmental stages and have a high degree of organization and high cellular diversity^58^. We generated cortical organoids from iPSCs (Fig. 5A) as previously described^7^. After embryoid body (EB) formation (days 3–5, Fig. 5Aa), when they began to brighten and have smooth edges, germ layer differentiation was initiated (days 5–7, Fig. 5Ab). At this point, the EBs were brighter at the periphery and exhibited radial organization, indicating neuroectodermal differentiation. After the EBs were embedded in Matrigel droplets, the tissue started to form buds (Fig. 5Ac) of expanded neuroepithelium, and ventricular-like cavities appeared. After 4 more days in culture, around day 14, the organoids were cultured further under agitation conditions on an orbital shaker, and brain cortical structures could be clearly identified using a phase-contrast microscope (Fig. 5Ad–f).

Typical forebrain regions, positive for FOXG1, were localized in the organoid periphery (Fig. 5Ba,b); these characteristics were maintained in culture up to 40 days (Fig. 5Bc) and beyond (data not shown). Sections from organoids at 20 days reveal RG cells positive for SOX-2 in the VZ zone^7,57^ as in human brain development; few post-mitotic neurons (positive for neuron-specific TUJ1) migrated outward within the forebrain structures to begin forming a pre-plate (Fig. 5Bd). After 1 month in culture (40 days), cortical organoids exhibited advanced differentiation of SOX-2^+^ progenitors and the expansion of TUJ1^+^ neurons, forming a thickened layer around the forebrain structures (Fig. 5Be). We observed TUJ1 (green) staining in the neurons that migrated from the RG in the VZ and SVZ to the CP region, which surrounded the forebrain structures within the cortical organoids. It is important to note the presence of oRG cells (characteristic of the human fetal brain, SOX-2^+^) in the OSVZ at both 20 and 40 days in culture (arrows, Fig. 5Bd,e).

To investigate whether cortical organoids can be used to test therapeutic agents that repair brain damage, the expression of NRG-1 and its receptor, ERBB4, was assessed. Endogenous NRG-1 (Fig. 5Ca,b) and ERBB4 (Fig. 5Cc,d) were not only expressed within the VZ and SVZ but also by cells in the IZ and PP areas at 20 and 40 days, respectively, in culture. These results suggest that neural stem and progenitor cells, as well as mature neurons, express not only endogenous NRG-1 but also its receptor, ERBB4.

### Heme induces structural disorganization and cell death in cortical organoids

**Figure 6:**
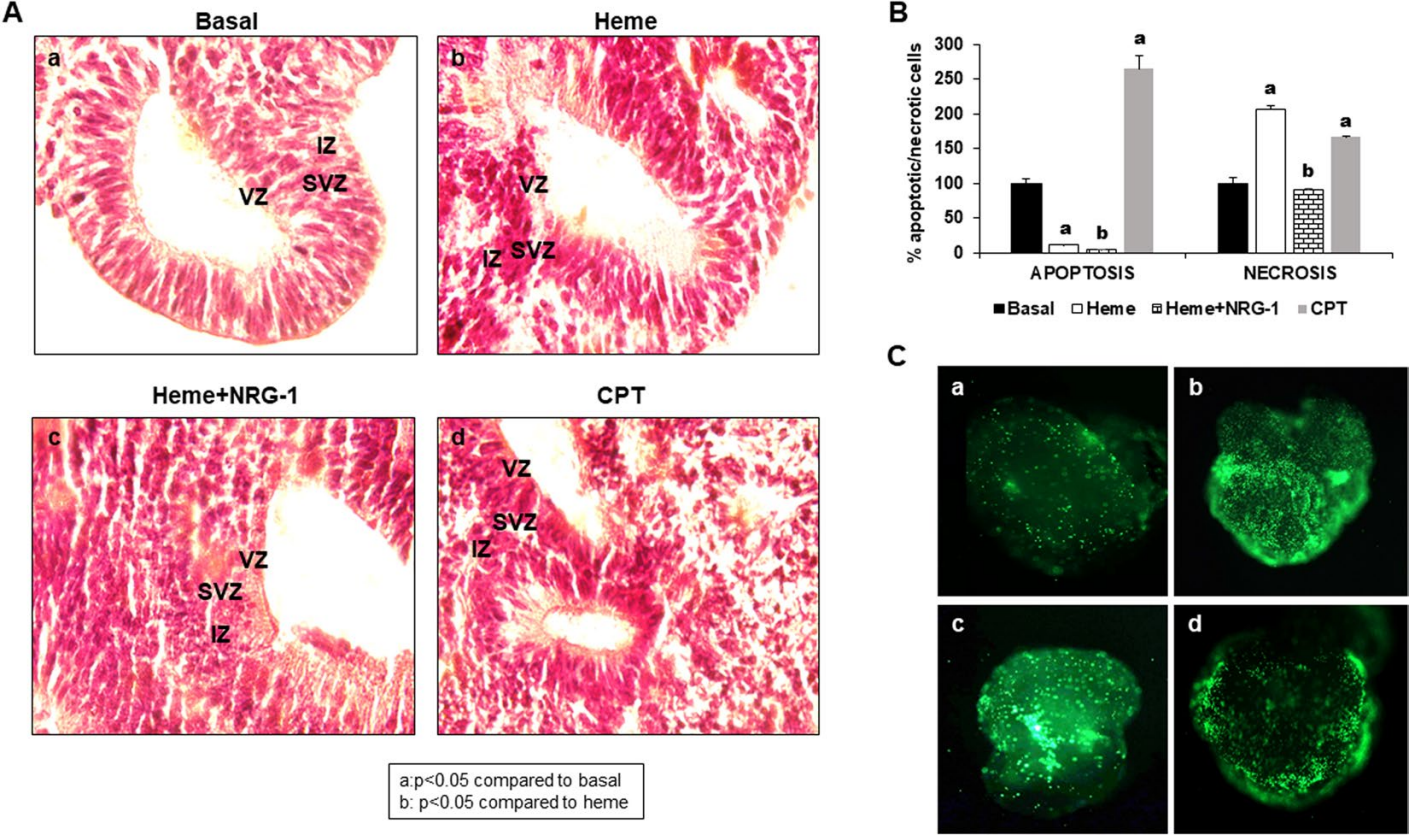
Heme modifies 20-day-old cortical organoid architecture by inducing early apoptosis and necrosis in its cells. Cortical organoids were treated with heme for 8 hours, and NRG-1 was then added for 18 hours. CPT treatment was used as a positive control for cell apoptosis. The results showed (**A**) that, compared to no treatment (a), heme induced the disorganization of cerebral organoid structure (b). NRG-1 improved the heme-induced lesion (c). CPT, used as a positive control for apoptosis, induced similar disorganization of cortical organoids (d). In addition, the apoptosis assay showed that heme increased cell necrosis in cortical organoids (**B**), while NRG-1 treatment reduced the injury. Representative images of cortical organoids (**C**) reinforcing the conclusion that heme had a toxic effect (b) compared to basal conditions, while NRG-1 had a protective role (d) compared to heme treatment (b). CPT also induced cell death compared to untreated condition (c) (a: p < 0.05 compared to basal conditions; b: p < 0.05 compared to heme treatment).

Cortical organoids have been previously used to model Zika virus (ZIKV) infection during human brain development^6,58^ and determine the causal link between fetal ZIKV infection and birth defects. The neocortex, which is disproportionally enlarged in primates and humans compared to mice, enables complex sensory activities and high cognitive functions that are impaired after malaria infection and other brain injury events linked to hemolysis. A key contributor to human neocortical growth is the expansion of SVZ progenitors, and defects in this process are thought to underlie a range of neurological disorders^59^. To investigate whether brain injury caused by malaria can be modelled in cortical organoids, we exposed organoids to heme, a by-product of erythrocyte hemolysis during malaria pathogenesis^54^ as well as other hemolytic disorders^60^. Hematoxylin and eosin (H&E) staining of 20-day organoids showed intact organization of the forebrain structures that contained the VZ and SVZ near a ventricular-like cavity (Fig. 6Aa). However, when organoids were exposed to heme and CPT, the VZ and SVZ (where neural stem cells reside), as well as IZ architecture, were disrupted (Fig. 6Ab,d). The addition of NRG-1 in the presence of heme improved the organization of the forebrain structures (Fig. 6Ac). To determine whether heme induces cortical organoid cell death, we measured heme-induced apoptosis and necrosis. Although early heme-induced apoptosis was lower (11.47%), necrosis was increased (205.98%) by more than two-fold compared to that observed under basal untreated control conditions (100%). Interestingly, NRG-1 treatment reduced heme-induced cell death (apoptosis decreased from 11.47% to 5.21%; necrosis decreased from 205.98% to 90.31%). CPT treatment, used as a positive control for inducing cell death, led to 264.81% apoptosis (2.64 times higher than that observed under basal conditions) and 166.47% necrosis (1.66 times higher compared to that observed under basal conditions) (Fig. 6B). Representative pictures of green fluorescence representing necrosis (Fig. 6C) showed that CPT and heme induced significantly higher levels of cell death (Fig. 6Cb,c) compared to that observed under basal conditions (Fig. 6Ca), while NRG-1 increased cell viability (Fig. 6Cd). It is important to note that NRG-1 increased cell viability not only in the central regions but also in the periphery of the organoids. Similar results were obtained with organoids at 40 days in culture (data not shown).

### Heme alters ERBB4/NRG-1, CXCL-10/CXCR3 and BDNF expression in organoid cells

**Figure 7:**
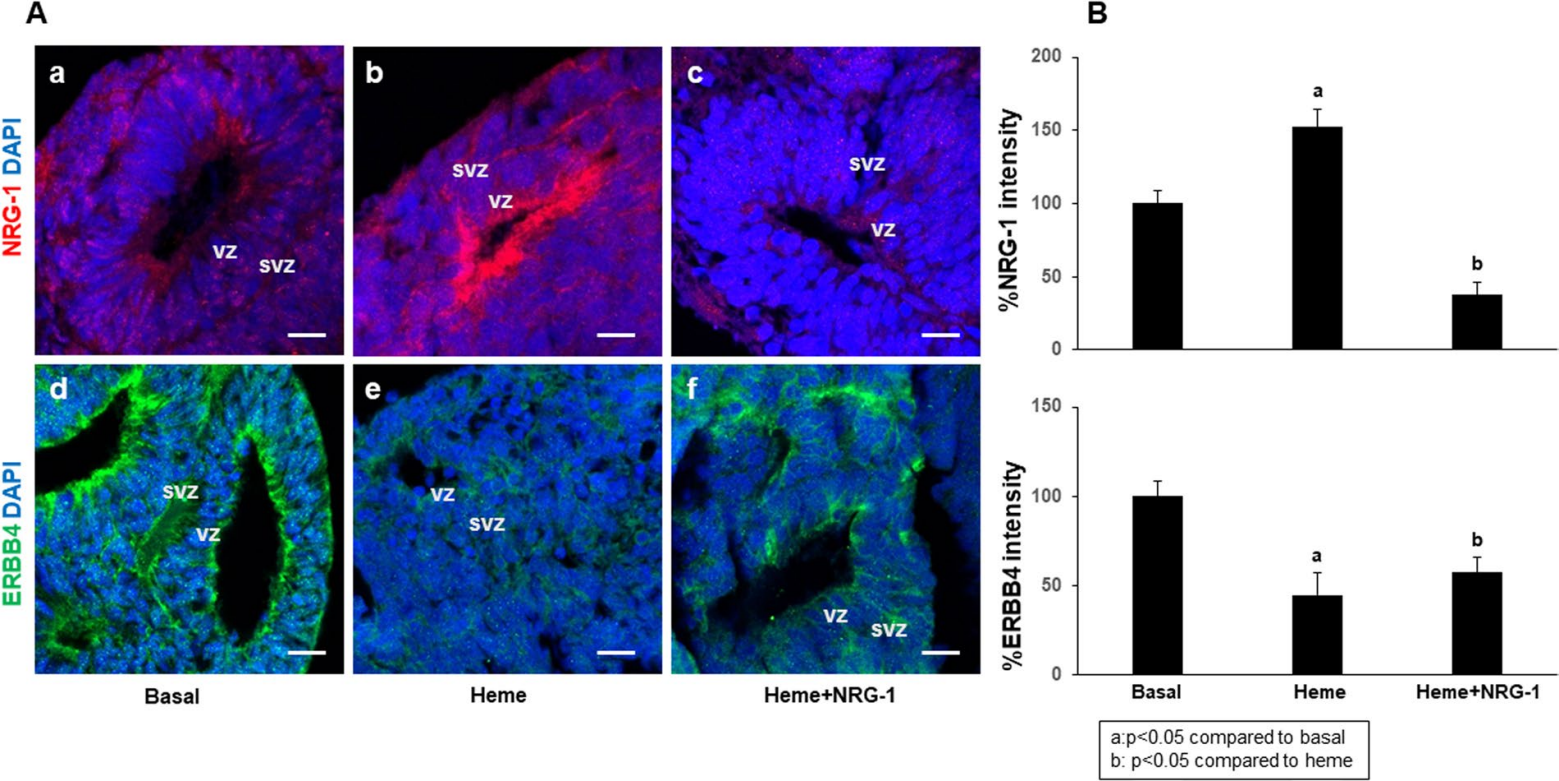
Heme induces NRG-1 and reduces ERBB4 expression in the forebrain structures of human organoids (20 days in culture). Cortical organoids were treated with heme for 8 hours, and NRG-1 was then added for 18 hours. The results showed that, compared to no treatment (a), heme induced NRG-1 expression. (b) The addition of NRG-1 to the treatment reduced its endogenous expression due to an improvement in cell injury. (c) ERBB4 expression decreased with heme treatment (e) compared to no treatment. (d) The addition of NRG-1 increased the levels of ERBB4 (f) compared to that induced by heme alone (e) but not compared to basal levels. (d) Scale bar: 20 µm.

**Figure 8:**
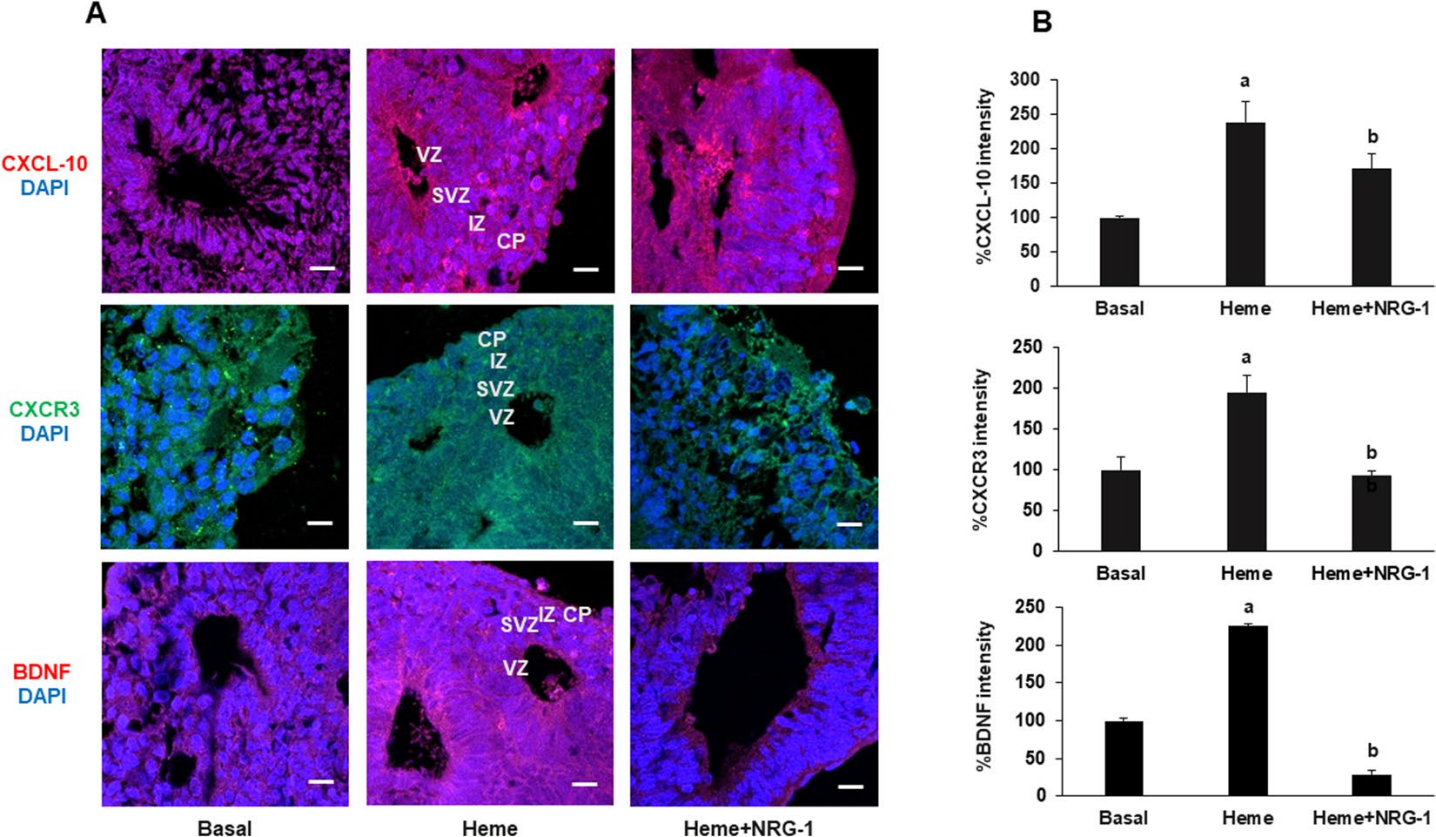
Heme induces inflammation in cortical organoids (40 days in culture). Forebrain organoids were treated with heme for 8 hours and then NRG-1 for 18 hours. Representative images of organoids under heme and NRG-1 treatment (**A**) showing the expression of the chemokine CXCL-10, its receptor CXCR3, and BDNF in the VZ, SVZ, IZ and CP of the cortical organoids. The quantification of the staining intensity showed that heme significantly increased the expression of CXCL-10, CXCR3 and BDNF, while NRG-1 significantly reduced this effect (**B**) in all zones of the forebrain organoids.

We investigated whether heme alters the ERBB4/NRG-1 axis (functionally active in brain injury) in cortical organoid cells. IHC showed that, compared to no treatment (Fig. 7a), 38 μM heme treatment increased NRG-1 expression especially in the cells of the VZ in the forebrain structures (Fig. 7b). In addition, compared to no treatment (Fig. 7d), heme decreased ERBB4 expression in all areas of cortical organoids (Fig. 7e). Treatment with NRG-1 (100 ng/ml) partially reversed the effects of heme, reducing the endogenous expression of NRG-1, mostly in the VZ (Fig. 7c), and increasing ERBB4 expression (Fig. 7f) in all structural zones in the organoids. At 40 days in culture, similar results were observed (data not shown). Consistent with previous results in 2D cell culture models and in ECM mouse models^29^, our present data suggest that heme affects the ERBB4/NRG-1 axis and that treatment with NRG-1 repairs heme-induced injury. Furthermore, the expression of CXCL-10 and its receptor, CXCR3, was increased by heme and decreased by NRG-1 in all organoid zones (Fig. 8A,B), in agreement with previous studies in 2D cell culture models suggesting that the induction of neuronal apoptosis by heme upregulates CXCL-10^61^. In addition, our results demonstrate that heme induces BDNF expression in forebrain organoids, while NRG-1 decreases this effect (Fig. 8A,B).

## Discussion

Until recently, direct studies of fetal and adult human brain development and disease pathogenesis were impossible due to ethical and technical reasons. In recent years, the emergence of iPSCs, the equivalent of embryonic stem cells, and 3D *ex vivo* culture systems called organoids have made these experiments possible^3^. Here, we have demonstrated that CD34+ cord blood-derived iPSCs (Figs. 2–4) and forebrain organoids (Figs. 5–8) can be utilized to study malaria-induced heme-mediated and other pathogen-induced brain injuries.

Free heme is a major component of hemoproteins (hemoglobin, myoglobin, cytochromes) and is deposited in tissues during acute or chronic pathologic conditions, causing organ and cellular injury^62^. During RBC invasion by *Plasmodium falciparum*, hemoglobin (Hb) is released into the circulation and dimerizes spontaneously. In the presence of reactive oxygen species (ROS), Hb is oxidized into metHb, leading to the release of heme. In the presence of ROS, cell-free heme promotes blood-brain barrier (BBB) disruption, promoting the onset of ECM^20^. Heme mediates oxidative stress, vascular apoptosis, and inflammation^63,64^ in tissues exposed to it due to general hemolysis. The effects of heme and ROS are attenuated by heme oxygenase-1 (HO-1), a stress-related enzyme that blocks the degradation of oxidation of cell-free Hb and the generation of free heme. HO-1 has been reported to play a protective role against HCM pathogenesis. Previous studies^65^ have shown that iPSCs depleted of HO-1 are more prone to oxidant-induced cell death and spontaneous differentiation, as evidenced by a decrease in OCT-4 expression. We previously reported that significant increases in circulating heme-induced HO-1 and CXCL-10 occur in ECM, HCM^61,66,67^ and *in vitro*^68^. Here, we showed that heme induced early and late apoptosis (Fig. 2) as well as spontaneous differentiation (Fig. 3) in iPSCs. Future studies will investigate whether treatment with HO-1 and other heme scavengers, protect against the toxic effects of heme in iPSCs and brain organoids. The expression of NRG-1 and its receptor, ERBB4, in iPSCs (Fig. 4) was correlated with reduced heme effects. The initial decline in the number of ERBB4+ cells at low heme doses and subsequent increase in response to increasing heme injury may be due to selection of heme-resistant iPSCs phenotype with increased ERBB4 expression. We have previously reported that neuroglial cells were more resistant to heme than endothelial cells^29^ and that HO-1 is produced as a result of heme-induced injury^28^. An elegant study showed that heme is highly toxic to cultured cortical neurons, leading to an increase in HO-1 expression. When exogenous HO-1 level was added, the neurons survived a formerly toxic concentration of exogenous heme^69^. Other studies have shown that HO-1 suppresses ECM incidence in a mouse model of cerebral malaria^20^. Future studies will determine whether HO-1, hemopexin and ERBB4 expression are associated with the iPSC resistance to heme-induced injury.

Many brain regions, including the cortex, have unique developmental trajectories that begin during fetal life and continue shortly after birth. In the neocortex, the proliferation of neural stem cells (radial glia) in the VZ continues with the migration of newly formed neurons to the cortical plate. Studies have shown^6^ that there is a correlation between developmental stages of the human fetal brain (especially the prefrontal cortex) and forebrain organoids. For example, day 26–54 organoid genetic profiles are similar to those of fetal cortex neurogenesis at 8–9 gestation weeks (GW), whereas the profiles of day 100 organoids are correlated with those of cortical areas at 17–35 GW, when the bulk of neurogenesis has occurred but radial glia maintain neurogenesis^70^. Cortical organoids (Fig. 5) expressed similar levels of ERBB4 and NRG-1 after 20 and 40 days in culture. Since brain organoids can be maintained for extended periods, they can model later events such as neuronal survival, maturation and degeneration beyond brain developmental stages^71,72^. Future studies investigating the effects of heme on astrocytes, oligodendrocytes, neuronal maturation, functional synapses and the induction of neural circuit dysfunction could be conducted using cerebral organoids after 6 months in culture^73^.

When humans are exposed to various toxic agents, the proliferation and migration of neurons to the cortical plate is disrupted. For example, in utero exposure to ethanol or methyl mercury leads to changes in cortical plate formation and neurological sequelae in childhood^74,75^. Events that occur early after birth, such as trauma or infections, can have a profound impact on the prefrontal cortex, which experiences the onset of a growth spurt in the first postnatal months and orchestrates attention and multi-tasking. Depending on the vulnerability of the cortex to toxic agents, children may experience various neurological sequelae in their childhood and adolescence. For example, intracranial bleeding after traumatic brain injury (TBI) leads to motor and cognitive dysfunction or impairment in children 4 years and younger, who have developing brains, compared to older children^76^. Using cortical organoids after various culture times, we were able to conduct studies to understand the effects of hemolysis by-products on their structure and function, and these results can be extrapolated to pre- and post-natal brain development after exposure to these by-products.

Cerebral organoids have been used to model microcephaly^3^, prenatal exposure to various drugs^77^, alcohol^78^, tobacco^79^, and infectious disease. Studies using cortical organoids have revealed that ZIKV causes disruption of cortical layers and slows their growth and the process of neurogenesis^80^. In addition, the caspase-3 inhibitors emricasan and niclosamide, an antihelmintic, have been found to be effective in limiting ZIKV replication and ZIKV-induced neural stem cell death^81^. We have developed, for the first time, cortical organoids that can be used to investigate heme-induced brain injury associated with malaria infection. The model can be used to study mechanisms related to HCM-associated brain swelling, inflammation, neuronal apoptosis, and factors mediating neurological sequalae in survivors. It can also be used in investigations involving malaria infection during pregnancy, which is associated with adverse effects, including fetal loss, premature delivery, intrauterine growth retardation and delivery of low-birth infants^82^. Our results show that heme reduces iPSCs survival and induces premature differentiation (Figs. 2 and 3), suggesting that brain organoids can be used to study the effects of hemolysis on fetal brain development not only during malaria infection but also during other hemolysis-associated diseases. The extravasation of blood during intracerebral hemorrhage induces neuronal damage in post-mortem studies^83^. Our results indicate that the exposure of organoids to heme disorganizes their architecture and induces apoptosis in neuronal cells localized at the periphery of the organoids (Fig. 6). These results correlate with the immunohistochemistry results obtained using post-mortem brain samples from patients with CM, where caspase-3 expression was increased in endothelial, neuronal and glial cells compared to control brain^84^. We previously demonstrated that heme induced endothelial cell apoptosis through Signal Transducer and Activator of Transcription 3 (STAT3) and its target gene Matrix Metalloproteinase 3 (*MMP3*) signaling^68^. In addition, heme upregulated the Tumor Protein p73 (TP73) levels in human brain endothelial cells *in vitro*; depletion of TP73 inhibited heme-induced apoptosis^22^. Furthermore, depletion of circulating endothelial progenitor cells during malaria pathogenesis is a function of heme-induced apoptosis mediated by CXCL-10 induction and toll-like receptor (TLR) activation^66^. The mechanisms by which heme induces neuronal apoptosis in HCM are not known and remain to be investigated using our organoid model.

We have previously reported that NRG-1 protects against heme-induced neuroglial apoptosis *in vitro*. Furthermore, in a mouse ECM model, NRG-1 serum levels increased in the immediate days after malaria infection, but then declined below the level in uninfected control mice when fatal brain damage occurred, suggesting inadequate protection of endogenous serum NRG-1 against severe late-stage ECM. NRG-1 expression in the brain cortex of mice with ECM increased. The addition of exogenous NRG-1 attenuated ECM associated mortality, suggesting that NRG-1 plays an important role in protecting the brain against ECM associated injury^29^. The expression of ERBB4 and its ligand, NRG-1, in the organoids encouraged us to test the hypothesis that the addition of exogenous NRG-1 can attenuate the deleterious effects of heme and reduce its apoptotic effects. The expression of endogenous NRG-1 and ERBB4 in the organoid cells was significantly altered by treatment with heme (Fig. 7). It has been shown that hemolysis induces inflammatory responses, including the induction of CXCL-10, in an ECM model^61^. Recent investigations demonstrate a tight correlation between higher serum BDNF concentrations and time to coma recovery and a reduced risk of disability in Ugandan children with HCM^26^. Furthermore, studies using animal models^85^ of traumatic brain injury have shown that BDNF expression persists around the injured area. The injury-induced upregulation of BDNF may restrict pro-apoptotic signaling following injury. Indeed, our results show that heme induced CXCL-10 and BDNF expression in forebrain organoids (Fig. 8). This can be explained by the cumulative neuroprotective effects of NRG-1 and BDNF that contribute to neuronal repair in damaged organoids. Although BDNF has a protective role against brain injury and is required for normal brain function across the lifespan, persistent enhanced BDNF brain levels may contribute to developmental, behavioral and cognitive impairment^86^. Future studies will determine the mechanisms mediating dysregulation of BDNF signaling in cerebral malaria pathogenesis.

Altogether, we conclude that the use of cortical organoids to assess heme mediated injury will enable a deeper understanding of the pathogenesis of cerebral and placental malaria as well as other hemolytic diseases and associated inflammatory mechanisms. In addition, iPSCs could be used not only to develop brain, but also other organoid types (hepatic, renal or pulmonary), that could be used to investigate how heme affects other organs. This model also opens up exciting opportunities for developing and testing new drugs to improve the outcomes of cerebral malaria worldwide.

## Materials and Methods

### Antibodies and reagents

Heme, radioimmunoprecipitation assay (RIPA) buffer and dimethyl sulfoxide (DMSO) were purchased from Sigma-Aldrich (St. Louis, MO). Camptothecin (CPT) was obtained from Selleck Chemicals (Houston, TX), while protease and phosphatase inhibitor cocktail were purchased from Thermo Fisher Scientific (Rockford, IL). Mouse β III tubulin [also called TUJ1; Covance (Burlington, NC)] and anti-mouse horseradish peroxidase (HRP)-conjugated secondary antibodies were purchased from BioLegend (San Diego, CA). A rabbit sex determining region Y box (SOX)-2 monoclonal antibody was purchased from Millipore (Chemicon) (Temecula, CA), while forehead box g1 (FOXG1), NRG-1 and ERBB4 rabbit monoclonal antibodies were purchased from Abcam (Cambridge, MA). CXCL-10 was obtained from PeproTech (Rocky Hill, NJ), CXCR3 was obtained from MyBiosource (San Diego, CA) and BDNF was obtained from Thermo Fisher Scientific.

### iPSCs and cerebral organoid culture

Human episomal iPSCs were purchased from Thermo Fisher Scientific (cat #A18945). The cells were obtained by reprogramming CD34+ human umbilical cord blood cells using a three-plasmid, seven-factor [SOKMNLT: SOX2, OCT4 (POU5F1), Kruppel-like factor 4 (KLF4), MYC (homologous to avian virus, myelocytomatosis oncogene), NANOG (homeobox transcription factor), LIN28 (regulates transition from pluripotency and committed cell lineages), and simian vacuolating virus 40 large (SV40L) T antigen] Epstein Barr nuclear antigen (EBNA)-based episomal system. This iPSC line is considered to be zero footprint because the reprogramming does not integrate into the genome. iPSCs were cultured under feeder-free conditions in dishes coated with Geltrex (Fisher Scientific). iPSCs were negative for mycoplasma, passaged using Versene (Fisher Scientific), cultured in Essential 8 Plus (Fisher Scientific) medium and incubated at 37 °C and 5% CO_2_. Cerebral organoids were developed as described by Lancaster *et al*.^3,87^. Briefly, on day 0, 9,000 iPSCs/well (less than passage 10) were dissociated using Accutase (Thermo Fisher Scientific) and then seeded in 96-well low attachment plates in embryoid body medium (Stem Cell Technologies) containing a Rho-kinase (ROCK) inhibitor (RevitaCell, Fisher Scientific). After 5 days, the embryoid bodies were cultured in induction medium (Stem Cell Technologies) to guide them towards a neuronal fate until day 10 and then expanded in Corning (Corning, NY) Matrigel Matrix droplets on a sheet of Parafilm. The droplets were allowed to solidify at 37 °C and were then transferred to 6-well ultralow attachment plates to expand the neuroepithelial tissues. After 4 days of growth in Matrigel, on day 14, the matrix was removed from the organoids by mechanical dissociation, and the organoids were grown in maturation medium from Stem Cell Technologies (Cambridge, MA) for 40 days on an orbital shaker obtained from Infors HT (Annapolis Junction, MD). Maturation medium changes were performed every 3–4 days.

### Measurement of iPSCs viability using CCK-8 assay after exposure to heme

The viability of iPSCs treated with heme and with or without NRG-1 was assessed by determining the number of live cells by colorimetry. The cell counting kit-8 obtained from Dojindo Molecular Technologies, Inc. (Rockville, MD) uses highly water-soluble tetrazolium salt, WST-8, which is reduced by dehydrogenase activity in cells to produce yellow formazan dye, which is soluble in tissue culture medium. The amount of formazan dye is directly proportional to the number of living cells. iPSCs (10,000 cells/well) were treated with heme (0, 10, 20, 30, 60, 90 μM) for 8 hours, then NRG-1 (100 ng/ml) for 18 hours, and then 10 μl of CCK-8 solution was added for 1 hour. Camptothecin (CPT) was used as a positive control for cell toxicity. The optical density of the colorimetric reaction was determined at 450 nm by a CytoFluorTM 2300 plate reader and CytoFluorTM 2300 v. 3A1 software (Millipore Co, Bedford, MA). The absorbance was plotted versus heme and NRG-1 treatment to determine cell viability.

### Flow cytometry analysis

To assess the expression of pluripotency markers (SOX-2, OCT-4, and SSEA-4), iPSCs were treated with heme (0, 10, 20, 30, 60, 90 μM or IC50 = 38 μM) for 8–18 hours and NRG-1 for 18 hours and analyzed using a multi-color flow cytometry stem cell kit (R&D Systems. iPSCs cultured in Geltrex-coated dishes were allowed to reach 70–80% confluence and then dissociated in a single cell suspension using Versene. The cells were incubated in fixation/permeabilization solution for 30 minutes and then washed and resuspended in permeabilization/washing buffer. Next, fluorochrome-labelled antibodies were added for 30–45 minutes in the dark at room temperature, and the cells were then washed with permeabilization/washing buffer and analyzed. The corresponding isotype-matching antibodies provided in the kit were used as negative controls and to determine nonspecific binding^88,89^. To investigate the number of cells that express the NRG-1 receptor following exposure to heme, iPSCs were incubated with an ERBB4 antibody and its recommended isotype control (Abcam), and then secondary antibody was added for 1 hour. To identify each cell population, the gating strategy was performed as previously described^90^. An electronic gate was placed to analyze 5,000 live cells in forward-and side-scatter for each sample. Then, a marker was placed at the limit of the negative control to assess all the positive events. Compensations to account for spectral overlap were performed for experiments that used more than one fluorophore. To assess heme-induced apoptosis, we used a FlowCollect Annexin Red kit (Millipore, Billerica, MA). iPSCs were treated with heme for 9 hours and then with NRG-1 for 18 hours. A single live cell suspension in assay buffer was incubated with Annexin V-CF647 working solution for 15 minutes at 37 °C. The cells were washed and resuspended in assay buffer, 7-AAD was added for 5 minutes, and the cells were analyzed. For apoptosis experiments, 10,000 events/sample were acquired using a flow cytometer (Guava system) and analyzed using unstained iPSCs exposed to complete medium only as a negative control. The results were analyzed using the InCyte program from GuavaSoft 3.1.1 (Millipore, Billerica, MA). All experiments were performed in triplicate.

### Immunohistochemistry staining

Immunohistochemistry (IHC) was used to assess the localization of various markers in cortical organoids grown in maturation medium supplemented with 2% Matrigel (to generate a basement membrane). Twenty- and 40-day-old organoids were treated with heme for 10 hours and then NRG-1 for 20 hours and fixed with 10% formalin, dehydrated with ethanol and embedded in paraffin blocks. Slices were made (10 μm), deparaffinized with xylene, rehydrated in a graded series of ethanol and double distilled water in a standard manner, and then stained with H&E. To detect protein expression, the sections were heated to unmask the epitopes and blocked with 10% goat serum, and specific primary antibodies were added overnight. Fluorescence staining was performed using fluorochrome-labelled secondary antibodies (Alexa-Fluor 488, Cy3, Sigma-Aldrich). The sections were covered with Vectashield mounting medium containing DAPI (H-1200) obtained from Vector Laboratories Inc. (Burlingame, CA). Images were collected by using a Zeiss 700 confocal laser scanning microscope (LSM) and a Zeiss 780 Elyra super resolution microscope, while images were captured using Zen black software (Carl Zeiss, Wetzlar, Germany). Tiled images were assembled using the Zen Tiles and Positions module. For brightfield imaging, an Olympus microscope (Irvine, CA) was used. To measure the intensity of ERBB4 and NRG-1 staining, ImageJ software was used^59^. After splitting the channels to obtain the red and green channels, the images were changed to grey scale, and then the fluorescence intensity was measured to determine marker expression. At least 3 forebrain structures from 3 separate organoids were measured per treatment type, and the mean intensity was used.

### Apoptosis/Necrosis determination in organoids

To assess early apoptosis/necrosis in cortical organoids, they were cultured for 20 and 40 days in maturation medium, and then treated with heme for 8 hours followed by NRG-1 for 18 hours. CPT was used as a positive control for apoptosis. We used the RealTime-Glo Annexin V Apoptosis and Necrosis Assay obtained from Promega (Madison, WI) following the manufacturer’s instructions. Briefly, the organoids were placed in 96-well low attachment plates, and then Annexin NanoBit substrate, CaCl2, necrosis detection reagent, Annexin V-SmBiT and AnnexinV-LgBiT were added. Heme-induced apoptosis was measured by using a plate reader [CytoFluorTM 2300 plate reader and CytoFluor 2300 v. 3A1 software (Millipore)] set for chemiluminescence, while heme-induced necrosis was detected by setting up the plate reader for green fluorescence (excitation 485 nm, emission 525 nm). The readings were normalized to the organoid volume, as measured by the formula (Lxw^2^)/2, where L is the large diameter and w is the small diameter of each organoid. L and were measured using ImageJ software. Green fluorescence images of heme-induced necrosis in cortical organoids were collected by using a Zeiss 700 confocal laser scanning microscope (LSM) (Carl Zeiss, Wetzlar, Germany).

### Western blot analysis

To assess the expression of proteins (NRG-1 and the receptor ERBB4) in cortical organoids, cell lysates from cortical organoids were prepared using RIPA buffer containing protease/phosphatase inhibitors. The protein concentration was determined in each sample using a Pierce BCA Protein Assay Kit (Thermo Fisher Scientific). Thirty micrograms of total protein from cell lysates were loaded on 8–15% SDS-polyacrylamide gels for Western blot (WB) analysis. After electrophoresis, the proteins were transferred to PVDF membranes. After blocking for 30 minutes in 5% skim milk-TBST buffer (TBS plus 0.1% Tween 20), the membranes were incubated with primary antibodies overnight at 4 °C and then incubated with horseradish peroxidase (HRP)-conjugated secondary antibodies and chemiluminescent substrate (Thermo Fisher Scientific). Specific antigen expression was evaluated using an Image Quant LAS400 system (GE Healthcare, Piscataway, NJ). GAPDH was used as the experimental protein loading control. The quantitative determination of proteins was performed using ImageJ.

### Statistical analysis and reproducibility

All experiments and determinations were performed in triplicate. Statistical comparisons were made using Student’s t test and ANOVA. The data are presented as the means + /− SEM. Values of p < 0.05 were considered statistically significant.

## Supplementary information

Supplimentary figure S1

## Data Availability

All materials, data and associated protocols will be made available to readers
