# Supplementary material for: Modelling heme-mediated brain injury associated with cerebral malaria in human brain cortical organoids
Source: Sci Rep. 2019 Dec 16;9:19162. doi: 10.1038/s41598-019-55631-8 (PMC6914785; doi:10.1038/s41598-019-55631-8)
Supplement: Supplementary file 1 — Supplimentary figure S1 [file 41598_2019_55631_MOESM1_ESM.pdf]

Modeling heme-mediated brain injury associated with cerebral malaria in human brain cortical organoids

Authors:

Adriana Harbuzariu[1]*, aharbuzariu@msm.edu

Sidney Pitts[1], spitts@msm.edu

Juan Carlos Cespedes[1], jcespedes@msm.edu

Keri Oxendine Harp[1], kharp@msm.edu

Annette Nti[1], anti@msm.edu

Andrew P Shaw[2], andrew.shaw@ibb.gatech.edu

Mingli Liu[1], mliu@msm.edu

Jonathan K. Stiles[1]*, jstiles@msm.edu

*Corresponding authors: Jonathan K Stiles and Adriana Harbuzariu

[1]Morehouse School of Medicine

720 Westview Dr

Atlanta, GA, 30310

Phone: 404-752-1585

[2] Parker H. Petit Institute for Bioengineering and Bioscience

Georgia Institute of Technology

315 Ferst Drive

Atlanta GA 30332 USA

Phone: 404-894-2000

SUPPLEMENTARY INFORMATION

SUPPLEMENTARY FIGURE S1

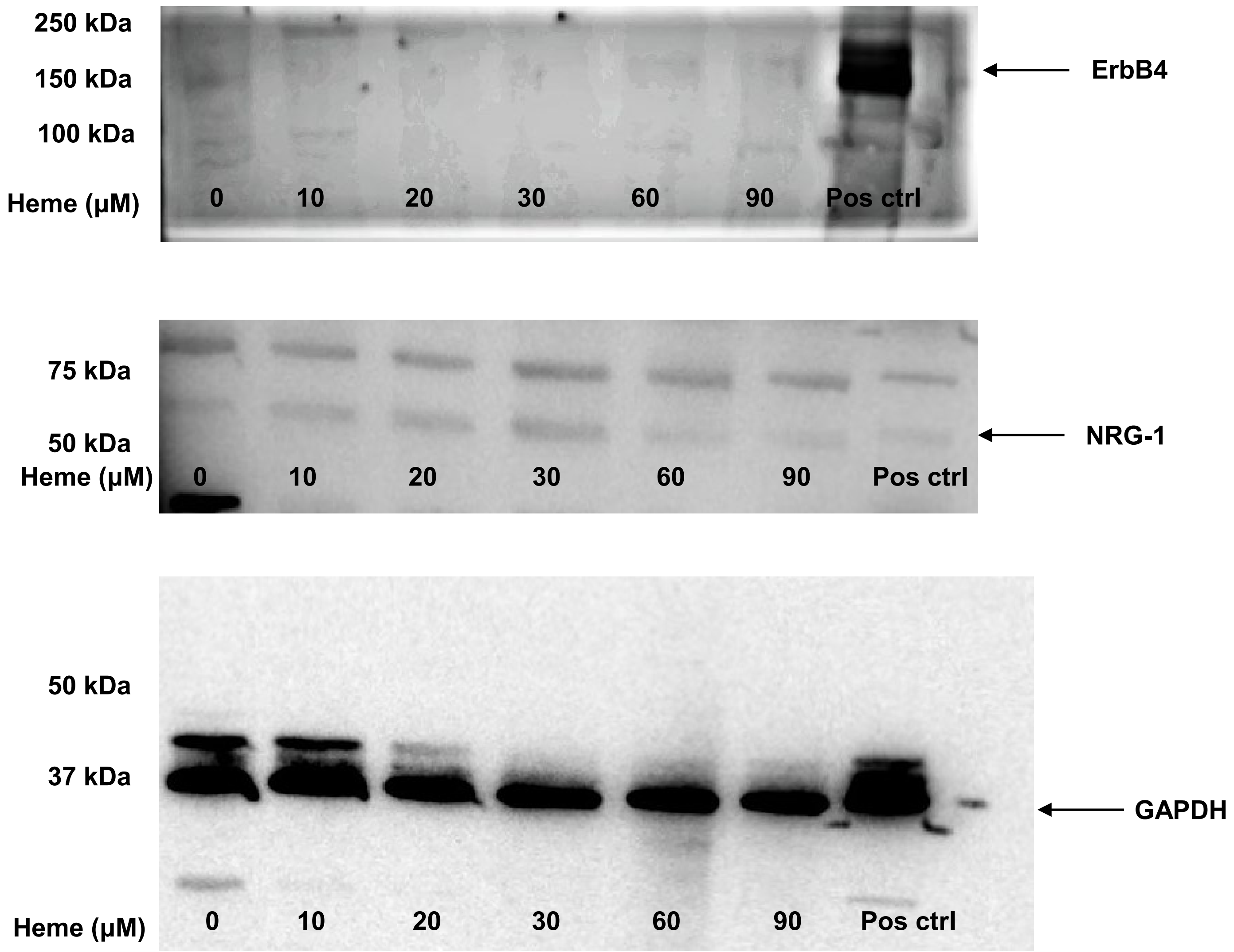

Figure S1. Full length western blots. Full length western blot for figure 4. The band images was acquired using Image Quant LAS4000 system. The quantitative determination of proteins was performed using Image J.
